# What Has Caused Desertification in China?

**DOI:** 10.1038/srep15998

**Published:** 2015-11-03

**Authors:** Qi Feng, Hua Ma, Xuemei Jiang, Xin Wang, Shixiong Cao

**Affiliations:** 1 Cold and Arid Regions Environmental Engineering Research Institute, Chinese Academy of Sciences, No. 320, Donggang West Road, Lanzhou, Gansu 730000, P.R. China; 2 Guangxi Hydraulic Research Institute, No. 1–5 Minzhu Road, Nanning, Guangxi, 530023, P.R. China; 3 School of Economics and Management, Beijing Forestry University, No. 35, Qinghuadong Road, Haidian District, Beijing, 100083, P.R. China

## Abstract

Desertification is the result of complex interactions among various factors, including climate change and human activities. However, previous research generally focused on either meteorological factors associated with climate change or human factors associated with human activities, and lacked quantitative assessments of their interaction combined with long-term monitoring. Thus, the roles of climate change and human factors in desertification remain uncertain. To understand the factors that determine whether mitigation programs can contribute to desertification control and vegetation cover improvements in desertified areas of China, and the complex interactions that affect their success, we used a pooled regression model based on panel data to calculate the relative roles of climate change and human activities on the desertified area and on vegetation cover (using the normalized-difference vegetation index, NDVI, which decreases with increasing desertification) from 1983 to 2012. We found similar effect magnitudes for socioeconomic and environmental factors for NDVI but different results for desertification: socioeconomic factors were the dominant factor that affected desertification, accounting for 79.3% of the effects. Climate change accounted for 46.6 and 20.6% of the effects on NDVI and desertification, respectively. Therefore, desertification control programs must account for the integrated effects of both socioeconomic and natural factors.

Drylands cover about 54 million km^2^, which amounts to 40% of the global land area, and are especially common in Asia and Africa, where they account for 58.5% of the world’s dryland area^1^. These regions have suffered from climate change, unfavorable hydrologic conditions, changes in vegetation composition, loss of soil services, and desertification; the combination of these effects has generated many adverse consequences, including sandstorms that threaten ecosystem services and human life^2^. In recent years, more and more of the sandstorms that form in desert areas have swept into modern cities in areas such as northwestern China, Africa, the western United States, and Australia^3^. In arid, semi-arid, and dry sub-humid regions, land degradation that results in a loss of vegetation cover is caused by several factors, including climatic change and human activities, and has been defined as *desertification*. As desertified areas expand, the area of livable habitat will decrease, and poverty will be exacerbated^4^. Desertification has become a crucial environmental problem at a global scale, and has begun to affect the survival and socioeconomic development of humankind.

Research has suggested that both climate and human activities play important roles in the process of desertification, which is complicated and includes complex interactions between human and natural factors (e.g., climate)^5^. Because of this complexity, past research has generally focused on either simple climate factors or on human activities rather than trying to account for both factors simultaneously. Some studies concluded that climate change affected the soil quality, vegetation cover, species composition, and hydrologic cycles in drylands, and has therefore led to expansion of the desertified area^6^,^7^,^8^. Others have argued that unsustainable traditional practices such as grazing, logging, and exploitation of underground water have created enormous pressures on ecosystems, leading to desertification^9^,^10^. Such human activities can eliminate the vegetation cover that protects the soil against erosion by water and strong winds^9^. However, without an understanding of how the interactions among the abovementioned factors affect desertification, it is difficult to reconcile the different research results. This creates a high risk of misunderstanding the current situation and adopting ineffective policies and programs to combat desertification^11^,^12^.

In northwestern China, desertification is a major ecological problem that has increasingly limited development of the local economy^13^. To control desertification, the Chinese government implemented a series of large-scale mitigation programs, including the Three Norths Shelter Forest Program and the Combating of Desertification Program^14^,^15^. These projects focus on increasing the vegetation cover by prohibiting grazing, planting trees and grasses, and constructing shelter forests to protect farmland against blowing sand. The total desertified area has decreased in many areas, but in others, desertification has continued to expand^16^. Several researchers have therefore questioned the effectiveness of solutions such as afforestation in drylands, and especially the practice of planting trees in arid areas that lack sufficient precipitation to sustain the trees in the long term, thereby requiring irrigation to ensure tree survival^11^,^17^,^18^.

Because the relative contributions of natural and human factors are unclear, the driving forces for desertification remain unclear. It is therefore urgently necessary to comprehensively study their interacting effects. Determining the relative contributions of natural and human driving forces to desertification would provide insights into the key mechanisms responsible for desertification, thereby leading to more effective responses. This approach is crucial because of the severity of the desertification problems that China faces and the large sums of money being spent to solve these problems. In the present study, we used the normalized-difference vegetation index (NDVI), obtained by means of satellite remote sensing, to monitor the progress of desertification in four regions of northwestern China: the Xinjiang Uyghur Autonomous Region, the Ningxia Hui Autonomous Region, Gansu Province, and the Inner Mongolia Autonomous Region. We then combined this data with climate and socioeconomic data to investigate the relative contributions of climate change and human activities to desertification and its reversal. Based on the results of this analysis, we discuss the key driving factors that are contributing to desertification and its reversal, and the lessons for planners of China’s ecological restoration strategy. This will provide important information on how to integrate the effects of climate change and human activities to develop solutions capable of mitigating the problems and promoting sustainable development in the regions that are facing desertification.

## Methods

To represent vegetation cover over large areas using the available long-term data, it is necessary to use satellite remote-sensing data. Of the available indicators, we chose NDVI because it has been used successfully for many years, by many researchers, and because it is a good proxy for the actual vegetation cover, especially in arid and semi-arid regions. The NDVI dataset used in this paper came from the AVHRR GIMMS group^19^ at a spatial resolution of 8 km. We used the 15-day maximum-value composites (MVCs) for the period from 1983 to 2006. We also obtained monthly NDVI MVC data from 2000 to 2010 from the Earth Observing System (EOS) satellites (http://glcf.umd.edu/data/ndvi/), at a spatial resolution of 500 m. We then converted the 2000 to 2010 NDVI data to use the same temporal and spatial resolution as the 1983 to 2006 NDVI data. To do so, we combined a 16 × 16 grid of EOS pixels to create a single AVHRR pixel, and calculated the weighted mean value of the 256 pixels in the grid to represent the overall value for both halves of the month (i.e., the two 15-day products). We used the mean of these two values to represent the monthly mean, and then selected the maximum value from monthly data to represent the year. We then performed simple linear regression to determine the relationship between the NDVI values in the AVHRR pixels and in the composite EOS pixels using data for the period of overlap from 2000 to 2006. The result was a moderately strong and statistically significant regression (*R*^2^ = 0.527, *p* < 0.05). We then used that regression to convert the EOS data from 2000 to 2010 into the corresponding AVHRR values. The result was a unified NDVI time series from 1983 to 2010.

We obtained the areas of desertification from national monitoring data in 1990, 2000, 2005, and 2010. We also obtained data on nine factors that potentially affected desertification, which we grouped into two categories: socioeconomic factors (the rural population, rural net income, farmland area, number of livestock, area of forest in which agriculture and grazing were prohibited, afforestation area, and the length of roads and railways) and climate factors (annual mean temperature and total annual precipitation). The socioeconomic data were obtained from the China Statistical Yearbook from 1983 to 2012^20^. The ecological restoration data were obtained from the China Forestry Yearbook from 1983 to 2012^21^. The meteorological data (annual mean temperature and total annual precipitation) were obtained from the China Climate Yearbook from 1983 to 2012^22^.

To understand how climate change and human activities have affected desertification, we established empirical models of the following form:


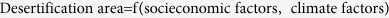



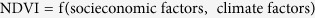


We analyzed panel data to identify the key factors, and compared their contributions to the area of desertification and to the vegetation cover (as represented by NDVI) during the study period. To avoid the impact of overlapping factors on the results, we employed the regression analysis module of version 11 of the STATA software (http://www.stata.com/) to calculate the regression coefficients for the relationships between all pairs of driving factors. The panel data model is:


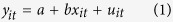


where *y*_*it*_ is the area of desertification or the vegetation cover for region *i* in year *t*, *x*_*it*_ is the corresponding socioeconomic factor, *u*_*it*_ is an error term, and *a* and *b* are regression coefficients. To account for the possibility of autocorrelation among the factors analyzed in our regression, we performed the Breusch-Godfrey LM test, and found no significant autocorrelation. Based on the results of an *F*-test, we selected a pooled regression model for calculating the effects of the abovementioned variables on the NDVI and area of desertification in four provinces (Xinjiang, Ningxia, Gansu, and Inner Mongolia) located in arid and semi-arid areas of China.

In the pooled model:


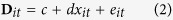


where **D**_*it*_ is the vector that contains the dependent variable (NDVI or the area of desertification) in study area *i* at time *t*; *c* is the intercept; *d* is the regression coefficient; and the random error was captured by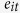.

We used the standardized regression coefficients to calculate the contribution of the different variables to the changes in NDVI or the area of desertification for the whole study region and for each province independently. The contribution is calculated as follows:


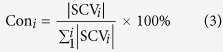


Where
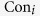
is the contribution of variable *i* to NDVI or desertification and 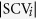 is the absolute value of the standardized coefficient value of variable *i*. Because the result of our calculations using NDVI as the dependent variable was not statistically significant for the individual provinces, we have only presented the results of our analysis of the area of desertification as a separate result for each province.

## Results

Table 1 summarizes the results of our analysis and the contributions of the driving factors to overall NDVI (for the four provinces combined) in arid and semi-arid regions of China. Based on the results for the pooled model, farmland area, forbidden area (the area in which grazing and agriculture were forbidden), cumulative afforestation area, and total annual precipitation were significantly positively related to NDVI change. Their contributions to NDVI change were 16.9, 5.7, 2.9, and 30.0%, respectively, which suggested that precipitation had the strongest effect on vegetation cover change, followed by the area of farmland. Livestock number and the length of roads and railways were significantly negatively related to NDVI change, accounting for 15.9 and 5.8% of the total effect, respectively. Both factors had an effect similarly strong to that of farmland area, but grazing (which is proportional to the number of livestock) had the strongest negative effect on NDVI change. The rural population, rural income, and mean annual temperature did not significantly affect NDVI change.

Table 2 summarizes the contribution of the driving factors to desertification change for the four regions. Livestock number, farmland area, road construction, and mean annual temperature were significantly positively related to the change in the area of desertification, accounting for 30.8, 21.9, 4.1, and 14.6% of the total effect, respectively, though the contribution of temperature was only marginally significant. As in the analysis of NDVI, livestock increased desertification (probably through vegetation loss caused by grazing). The rural population (10.6%), rural net income (7.8%), area in which grazing and agriculture were forbidden (4.2%), and total annual precipitation (6.0%) were significantly negatively related to the change in the area of desertification, though the contribution of annual precipitation was only marginally significant. However, the afforestation area was not significantly correlated with desertification dynamics, which suggests that afforestation did not contribute to desertification control in the long term.

**Figure 1:**
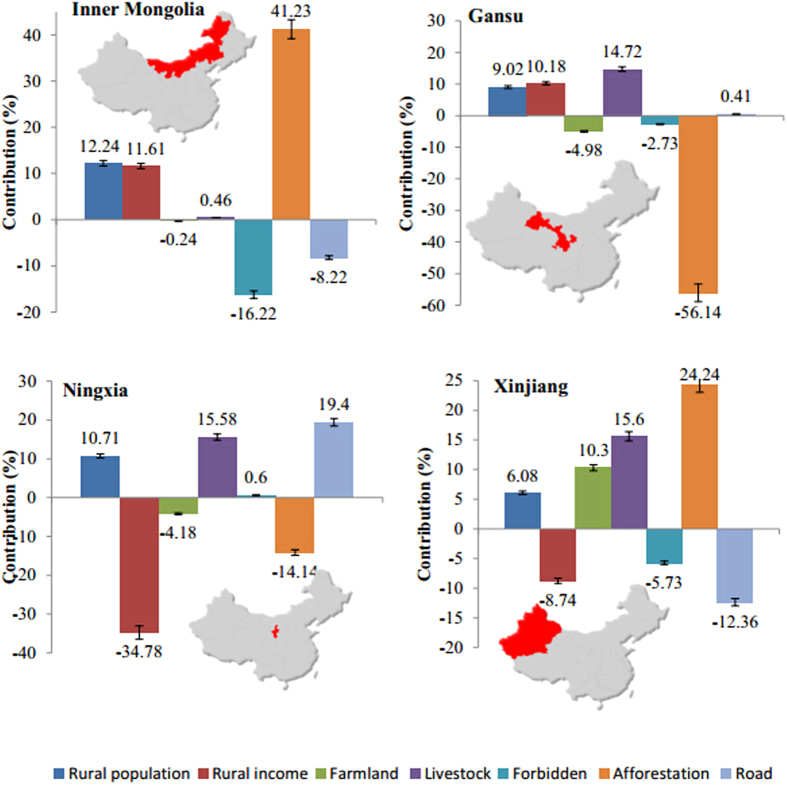
Contributions (%) of the driving factors to changes in the area of desertification based on the results of the regression analysis. The data have been broken down for the four study areas, and are based on the relationships among the values of the driving forces. “Forbidden” represents the area of forest in which agriculture and grazing were prohibited, and “Road” represents the length of roads and railways. We created this figure in using ArcGIS 10.0 for maps.

The contribution of each variable to the change in the area of desertification in the four provinces (Fig. 1) differed among the provinces, suggesting that the drivers are specific to the context of each province. The rural population and livestock numbers in all four provinces were positively related to increases in the area of desertification, but the other variables showed different effects in different regions. In Inner Mongolia and Xinjiang, afforestation was the most important contributor to desertification, accounting for 41.2 and 24.2% of the total effect, respectively, whereas afforestation resulted in restoration in Gansu and Ningxia, accounting for 56.1 and 14.1% of the total effect, respectively. Remarkably, forbidding agriculture and grazing was associated with decreased or only slightly increased desertification in all four regions, which means that this approach is potentially effective for ecological restoration. The effects of road construction also differed among these provinces. For Inner Mongolia and Xinjiang, road construction decreased desertification, possibly because vegetation restoration and irrigation projects tend to be associated with additional road construction in arid regions such as Inner Mongolia and Xinjiang.

## Discussion

Our results demonstrate that the combination of significant rural socioeconomic factors and significant climatic factors had an important effect on vegetation cover (as measured by NDVI) and on the area of desertification in the four arid and semiarid areas of China that we studied. In these regions of China, the high level of human activities and the strength of the associated impacts result from cultivation, grazing, destruction or harvesting of herbaceous vegetation, and logging forests to produce firewood and rural construction materials. The local crops are mainly wheat, potato, and cotton, and their areas can be detected using NDVI data during their growing seasons; this may partly explain why the area of farmland had a positive effect on NDVI (Table 1). The expansion of crops can potentially increase the vegetation cover, but this increase is temporary; for most crops, the soil remains uncovered during the fallow season. If the farmland is abandoned without the implementation of effective protection of the soil, desertification will accelerate due to increased erosion by the wind^22^. As the rural population decreased during the study period^19^, the pressure from the demand for land should also have decreased. Although the rural population is often considered to be a major driving force for environmental damage, it is also an important force for managing farmland and grazing to avoid damage to vegetation.

Rural poverty alleviation is as important as desertification control and ecological restoration^23^,^24^. Even though the rural net income gradually increased every year during the study period^19^, it has been difficult to raise farmers and herders out of poverty. The first problem is that the harsh environment and the large population of impoverished rural residents make the income from traditional farming highly vulnerable to natural disasters such as drought and to fluctuations of market prices^25^,^26^. Second, the study region’s simple economic structure makes it difficult to provide alternative forms of employment that would improve rural incomes. Third, the burden on residents of national efforts to control desertification is too heavy. The subsidies provided by the government to compensate residents for grazing and farming prohibition are less than the increasing cost of production and household expenses that result from these government policies^22^,^27^.

For rural residents in the arid and semiarid areas of China, their income mainly comes from the land, and the harshness of the environment (particularly the lack of water) means that earning this income jeopardizes the ecological environment; in particular, it can lead to soil erosion and an expansion of desertification. As our analysis revealed, the contribution of livestock is considerable. This is likely to be because poor communities must increase their livestock numbers to provide income or a food source; as this occurs at the expense of the environment, it exacerbates desertification. Our study confirmed that grazing and farmland expansion were important drivers of land degradation for all four provinces. Although the policy that restricts grazing has been implemented for almost a decade, livestock remain a significant cause of desertification. Analyses such as the present study reveal important impacts of such socioeconomic factors on desertification, and government ecological restoration policy that is implemented to counteract desertification must account fully for the economic losses of local residents under new policies by providing adequate subsidies or alternative means of employment. Without such efforts to protect the livelihood of these people, they have no ability to protect their environment, even when they understand that their activities are causing significant damage to that environment^24^.

The arid and semiarid areas of China, which occupy half of China’s total land area, are likely to face increasing stress from climate change, which will exacerbate existing water shortages and place additional stress on vegetation communities that are already being stressed by regional warming^28^. In our study, rural socioeconomic factors had a slightly stronger effect than climate factors on NDVI, accounting for 53.4% of the total effect. However, the statistically significant climatic factor (total annual precipitation) had a cumulative effect (30%) similar to that of the statistically significant socioeconomic factors (27.2%) on vegetation restoration. The cumulative values for these two groups of factors were sufficiently close that climate change and human activities appear to have accounted for similar proportions of the overall changes in vegetation cover.

For the factors that controlled desertification, the contribution of socioeconomic factors was clearly dominant (79.4% of the total effect for all factors, versus 79.3% for only the significant factors; Table 2); natural factors (temperature and precipitation) accounted for a much smaller portion of the total effect. Thus, human activities have had the dominant effect on desertification, but climatic factors have been significant, and their effect will become increasingly significant as a result of the warmer and drier climate produced by global warming. Based on the warming trend in northwestern China^28^, agriculture, grazing, and afforestation, which are sensitive to climate, must be carefully assessed to predict the effects of changes in temperature and precipitation on their impacts.

Some researchers have questioned the effects of planting trees in restoration projects to control desertification because this approach has not performed as well as expected^11^,^27^. In the present study, our results show that the contribution of the area of forest in which agriculture and grazing were prohibited (the “forbidden” area) and of the afforestation area averaged only 4.3% of the total effect for NDVI and only 2.1% for the area of desertification. The complexity of ecosystems and the even more complex interactions between humans and nature^29^,^30^ mean that the simplistic solution of planting trees in arid regions is unlikely to be a broadly applicable way to restore degraded dryland ecosystems. For example, planting trees (which often have low water-use efficiency) in arid regions often requires supplemental irrigation, which exacerbates the stress on an already limited water resource^12^, increases evapotranspiration, and can even exacerbate soil erosion if the trees outcompete herbaceous vegetation for water, leading to decreased vegetation cover at the soil surface^5^,^18^. The lack of a significant impact of the cumulative afforestation area on desertification means that the positive effects of afforestation in the short term may be compromised by the negative effects of afforestation on water availability in the long term. The low survival rate of the trees^11^ can also represent a large waste of labor and money. In many arid regions, restoration using herbaceous vegetation will produce better results than using trees or shrubs^31^. Although China’s huge national ecological restoration policies, such as the Three Norths Shelterbelt Project, have improved the vegetation cover in many areas, the potential risks should receive more attention from policy makers and restoration managers. The large variation among regions shown in Fig. 1 provides additional support for this recommendation, since these results demonstrate the strong effect of differences in local conditions.

The effects of ecological restoration in desertified areas result from the interactions among multiple factors. In the arid and semiarid areas of China, our results show roughly comparable impacts of socioeconomic factors and climate change for NDVI but stronger socioeconomic effects on the area of desertification. However, the strengths of the impacts varied widely among the four parts of our study area. This means that it will be difficult to fully understand the driving forces responsible for desertification in China without understanding the unique context of each region, and that monolithic policies will work less well than adopting policies that address the most significant driving factors for each region. It is evident that the relationships between the driving factors and the changes in NDVI and the area of desertification are complicated. The most important driving factors varied among the regions (Fig. 1). Thus, despite the significant impacts of several driving factors for the overall study area (Tables 1 and 2), policy development must be based on a careful examination of each individual region using a method similar to the one developed in the present study to identify the most significant driving factors for that region. Only then will it become possible to develop solutions that attack the most relevant problems. This finding has significant implications for achieving ecologically sustainable development in the degraded lands of China.

One limitation of our study is that we did not account for the sociological factors that underlie the human factors that we included in our analysis. This suggests that interdisciplinary research will be required to fully understand the sociological factors and their interaction with natural factors so that appropriate policy measures can be developed to focus on those factors and interactions. Although we made an effort to control for the uncertainty in our analysis by including the error term *u*_*it*_ in the regression analysis, an additional area for future research will be to more precisely determine the error and uncertainty that are associated with the socioeconomic factors. This will allow future researchers to better control this error term and more precisely estimate the impacts of individual factors. In addition, the regression relationship we developed will need to be validated by means of a pilot study that provides more detailed information. The results of our NDVI analyses are not surprising, since similar conclusions have been published by many researchers. For example, overgrazing and road construction are likely to result in decreased vegetation cover, as Li and Li^32^ found in their study of a government policy to end the traditional nomadic culture; the resulting sedentarization contributed to overgrazing, which in turn led to grassland degradation. Deng *et al.*^33^ found that road construction may lead to ecosystem degradation in high-quality grassland.

Although our method of identifying the contributions of each driving factor is defensible for providing a broad overview, it is likely that there is a better method for this kind of analysis that will support more precise analyses for individual regions. This method should be identified in future research to improve the ability of this research to support restoration planning.

## Additional Information

**How to cite this article**: Feng, Q. *et al.* What Has Caused Desertification in China? *Sci. Rep.* **5**, 15998; doi: 10.1038/srep15998 (2015).

## Figures and Tables

**Table 1:** Regression results for the relationship between the driving factors and NDVI, and the contribution of each factor to the changes in NDVI from 1983 to 2010.

	Pooled	Standard error	Contribution (%)
Rural population (×10^9^ persons)	0.249	−0.25	0.60
Rural net income (×10^6^ RMB)	12.46	−1.55	5.66
Farmland area (×10^6^ ha)	0.0190*	−3.25	16.93
Livestock number (×10^9^ head)	−0.142*	−2.91	15.91
Forbidden area (×10^6^ ha)	0.0394*	−3.34	5.66
Cumulative afforestation area (×10^6^ ha)	0.00747*	−2.70	2.90
Length of roads and railways (×10^6^ km)	−0.598**	−3.44	5.78
Mean annual temperature (°C)	1.106	−1.92	16.61
Total annual precipitation (mm)	0.733**	−14.20	29.95

Values represent the overall results for the four study regions (Xinjiang, Ningxia, Gansu, and Inner Mongolia); results for individual regions were not statistically significant, and are not shown. “Forbidden area” represents the area in which grazing and agriculture were forbidden. Sample size: *n* = 108.

Significance levels: **1%, *5%.

**Table 2:** Regression results for the relationship between the driving factors and the area of desertification, and the contribution of each factor to the changes in the area of desertification from 1990 to 2010.

	Pooled	Standard error	Contribution (%)
Rural population (×10^9^ persons)	−177.5**	−3.90	10.55
Rural net income (×10^6^ RMB)	−690.1*	−2.74	7.80
Farmland area (×10^6^ ha)	0.987**	−3.61	21.87
Livestock number (×10^9^ head)	11.050**	−7.24	30.80
Forbidden area (×10^6^ ha)	−1.177*	−2.89	4.20
Cumulative afforestation area (×10^6^ ha)	−0.00792	−0.09	0.08
Length of roads and railways (×10^6^ km)	16.90*	−3.05	4.06
Mean annual temperature (°C)	39.20^+^	−2.14	14.64
Total annual precipitation (mm)	−5.898^+^	−2.33	6.00

Values represent the overall results for the four study regions (Xinjiang, Ningxia, Gansu, and Inner Mongolia); results for individual regions were statistically significant, and are shown in Fig. 1. “Forbidden area” represents the area in which grazing and agriculture were forbidden. Sample size: *n* = 108.

Significance levels: **1%, *5%, ^+^10%.
